# Incidence of sarcoid-like reaction demonstrated on 18F-FDG PET-CT in head and neck cancer patients

**DOI:** 10.1186/1470-7330-14-S1-P5

**Published:** 2014-10-09

**Authors:** LI Sonoda, A Lakhani, S Ghosh-Ray

**Affiliations:** 1 Paul Strickland Scanner Centre, Mount Vernon Hospital, London, UK

## Aim

Sarcoid-like reaction (SLR) is a well-known benign cause of nodal FDG-uptake in cancer patients and needs to be differentiated from active nodal metastatic disease. This study aimed to demonstrate the prevalence of sarcoid-like reaction in head and neck cancer (HNC) patients demonstrated on 18F-FDG-PET-CT.

## Methods

A 6-year-period (2008-2013) retrospective analysis of 729 PET-CT scans (308 patients) of HNC was performed. Pre and post-treatment scans were compared and cases with persistent mediastinal/hilar nodal uptake despite therapeutic response elsewhere were further analysed and clinically followed up. Final diagnosis was reached by biopsy, clinical and imaging follow-up.

## Results

SLR was diagnosed in 8/308 (2.6%) HNC patients. Of which 3 had mediastinal nodal uptake only, 2 had bilateral hilar nodal uptake only and 3 had both mediastinal and hilar uptake. All the 8 patients had primary HNC and deep cervical lymphadenopathy but no distant metastases, hence misinterpretation of SLR would have upstaged as M1 (metastasis positive). 4 cases were identified on scan for restaging of suspected recurrence, and 4 were for primary tumour staging. The mean SUVmax of SLR was 6.9 (range 2.4-9.6), and the mean SLR nodal size was 1.6cm in short axis (range 0.6-2.4cm), with no significant difference compared to active nodal diseases (P=0.33 and 0.23).

## Conclusion

SLR was seen in 2.6% of HNC patients at 18F-FDG PET-CT examinations. 18F-FDG PET-CT plays a significant role in the management of HNC, and it is important to be aware of a possibility of SLR in order to avoid false-positive interpretation of metastasis.

